# Increasing trends in hepatitis E hospitalisations in Spain, 1997 to 2019

**DOI:** 10.2807/1560-7917.ES.2024.29.43.2400118

**Published:** 2024-10-24

**Authors:** María Guerrero-Vadillo, Marina Peñuelas, Rocío Carmona, Inmaculada León-Gómez, Carmen Varela

**Affiliations:** 1 Programa de Doctorado en Ciencias Biomédicas y Salud Pública, Universidad Nacional de Educación a Distancia (UNED), Madrid, Spain; 2 Departamento de Enfermedades Transmisibles, Centro Nacional de Epidemiología (CNE), Instituto de Salud Carlos III (ISCIII), Madrid, Spain; 3 CIBER de Epidemiología y Salud Pública, Instituto de Salud Carlos III (CIBERESP, ISCIII), Madrid, Spain

**Keywords:** hepatitis E virus, viral hepatitis, temporal trends, epidemiology, hospitalisation, electronic records, epidemiological surveillance

## Abstract

**Background:**

Hepatitis E, a viral hepatitis caused mainly by the ingestion of raw or undercooked food, is not a notifiable disease in Spain.

**Aim:**

To analyse the temporal trends, epidemiological characteristics and factors associated with severe disease from hepatitis E hospitalisations in Spain from 1997 to 2019.

**Methods:**

Hospitalisation records were obtained from the Spanish National Hospital Discharge Database. Temporal trends and seasonality were analysed by Poisson regression in years 1997–2015 and 2016–19, given changes in hospital discharge databases. Multivariate logistic regression was used to identify factors associated with severe disease.

**Results:**

Hepatitis E hospitalisation incidence increased from 0.22 cases per 1,000,000 inhabitants in 1997 to a maximum of 2.95 in 2018. Seasonality was observed during 2016–19 period, with more cases in the second and third quarters of the year. The incidence was higher in men vs women, and in the population aged over 40 years. Factors independently associated with death were age ≥ 50 years (adjusted odds ratio (aOR): 2.43), chronic liver disease (aOR: 4.29), HIV infection (aOR: 3.00) and hepatitis B/C (aOR: 2.11).

**Conclusions:**

Hepatitis E hospitalisations have increased in Spain in recent years, being more severe in cases with older age, chronic hepatic diseases and HIV infection. A greater incidence in men over 40 years and a possible seasonality were observed. Further studies are needed to assess the seasonality, geographical distribution and impact of the disease to guide public health actions for prevention and control.

**Key public health message**

**What did you want to address in this study and why?**

Hepatitis E is a liver disease that may be acquired by eating contaminated food like undercooked or raw pork products. Incidence has increased in Europe in recent years. In Spain, hepatitis E is not reported at national level, so temporal trends and characteristics of cases are not well defined. We wanted to analyse hepatitis E hospitalisations over the last 2 decades using electronic hospital records.

**What have we learnt from this study?**

We observed an increasing trend in the number of hepatitis E hospitalisations in Spain, especially in men over 40 years. Moreover, we observed more cases in the months of April to September. Patients with certain conditions, such as aged > 49 years, chronic liver disease, HIV infection and hepatitis B or C were more likely to experience severe outcomes with hepatitis E.

**What are the implications of your findings for public health?**

Our analysis of hospitalisation electronic records suggests that the disease may be particularly severe in some population groups. Differences among the Spanish regions and throughout the year may reflect risk factors that should be addressed.

## Introduction

Hepatitis E is an inflammatory liver disease caused by infection with hepatitis E virus (HEV). The disease has an incubation period of 2 to 6 weeks, and is frequently self-limiting, with symptoms of abdominal pain, malaise, vomiting, anorexia and hepatomegaly that last several weeks. While most acute HEV infections are asymptomatic [1], extrahepatic manifestations of the disease have also been described, such as neurological, haematological and renal symptoms [2,3]. Several factors related to severe disease and chronicity have been identified, such as chronic liver disease and immunosuppression [4].

Of the HEV 4 genotypes mainly affecting humans, genotypes 1 and 2 (HEV1–2) are mainly found in low-income countries where the virus is usually transmitted by faecal-oral route and is an important cause of epidemics. Genotypes 3 and 4 (HEV3–4) are present in high-income countries, including Europe, where the disease is considered an emerging zoonosis caused by the ingestion of contaminated food, i.e. mainly undercooked pork products and game meat, as well as shellfish; it is usually presented as sporadic cases or small clusters linked to the exposure to a common food. There are also reported cases transmitted by blood products and solid organ transplantation [1,5]. In Europe, there has been an increased number of HEV autochthonous cases, from 514 cases in 2005 to 5,617 cases in 2015 [6]; males and those above 50 years old are the most affected populations, and HEV3 is the genotype most frequently detected [7]. 

Hepatitis E surveillance in European Union/European Economic Area (EU/EEA) is heterogeneous, and in 2015, only 20 of 30 countries had specific systems, either voluntary or mandatory; in 15 of 20 countries, a national system was established, whereas 5 of 20 countries had reference laboratory surveillance, blood centre surveillance or sentinel surveillance. Case definitions used across countries were also not homogeneous, and they combined different clinical, epidemiological and laboratory criteria [7]. Consequently, the epidemiology of hepatitis E in Europe may not be entirely understood. 

In Spain, the notification of hepatitis E outbreaks is mandatory; nonetheless, only two outbreaks (each with two cases) have been notified since 2010. By contrast, hepatitis E cases are not subject to mandatory notification to the national level, and incidence rates and temporary trends are not well defined. Epidemiological characteristics of hospitalised cases, such as age, sex, comorbidities and mortality are also not well known. This study aimed to analyse the temporal trends, epidemiological characteristics and factors associated with severe hepatitis E in Spain, using electronic hospital records from 1997 to 2019.

## Methods

### Study setting and population

Hospitalisations caused by hepatitis E from 1997 to 2019 were obtained from the hospital discharge records in the national health system (Minimum Basic Data Set, CMBD in Spanish). The system, which was created in 1987, is the main database of morbidity and patient care process of the hospitals in Spain and contains information related to discharge records from both public and private acute care hospitals in the entire country. Since 2014, the CMBD includes more than 92% of discharges from acute hospitals in Spain [8]. Since 2016, the system also includes data from major outpatient surgeries, day hospitals, home-based hospitals, outpatient procedures of particular complexity and emergencies [9]. All episodes which contain any hepatitis E diagnosis code (primary or secondary codes, i.e. if it was or was not the main cause of hospitalisation) were included in the database. The study includes two temporal periods (Period 1: years 1997–2015; and Period 2: years 2016–19) because of the implementation in 2016 of the International Statistical Classification of Diseases and Related Health Problems (ICD) new edition.

### Data collection

Diagnosis codes from discharge records were coded according to the International Classification of Diseases Ninth and Tenth Edition (ICD-9 and ICD-10) for Period 1 and Period 2, respectively [10,11]. Hepatitis E hospitalisations were defined as any record with hepatitis E diagnosis codes 070.43 (hepatitis E with hepatic coma) or 070.53 (hepatitis E without mention of hepatic coma) for Period 1, and B17.2 (acute hepatitis E) for Period 2.

From each episode, the following data were extracted: age, sex, region of residence of the patient, hospitalisation date, length of stay (LOS) (in days), extrahepatic manifestations of HEV infections (defined as neurological manifestations, e.g. neuralgic amyotrophy, Guillain–Barré syndrome and mononeuritis multiplex; thrombocytopenia; acute pancreatitis; and renal manifestations) and comorbidities (defined as chronic liver disease, diabetes, alcohol use, malignant neoplasms, hepatitis B and/or C, HIV infection and transplant). The ICD-9 and ICD-10 codes used to define HEV infection, extrahepatic manifestations and comorbidities are provided in Supplementary Table S1. Severe hepatitis E was defined as prolonged hospitalisation (LOS more than 6 days), multiple episodes (two or more hospital admissions per patient) or death.

### Data analysis

Multiple episodes for the same patient were collapsed into a single hospitalisation: hospitalisation date was defined as the first date of contact with the hospital and LOS was the sum of all days in each episode. Extrahepatic manifestations, comorbidities and factors of severe disease were also collapsed into a single hospitalisation if they were presented in at least one of the episodes.

Annual hepatitis E hospitalisation incidence per 1,000,000 inhabitants (overall, by sex, and by age groups: 0–19, 20–39, 40–64 and ≥ 65 years old) were calculated using the total Spanish population as the denominator, extracted from the National Institute of Statistics (INE in Spanish) [12]. In addition, for each region of the country, we calculated the hospitalisation incidence per 1,000,000 inhabitants, overall and by period, by dividing the total number of cases by the sum of the populations of each year (also extracted from INE).

The number of hepatitis E hospitalisations was analysed separately by periods (1997–2015 and 2016–19) using a generalised linear model (Poisson regression) to assess temporal trends and seasonality, using sine and cosine functions, and quarter as time unit. Incidence-rate ratios (IRR), 95% confidence intervals (CI) and p values were calculated. Annual trends regarding the annual median age of cases and the annual median LOS were measured by simple linear regression, using the year as the independent variable. 

Multivariate logistic regression was applied to identify demographic and clinical factors associated with severity (prolonged hospitalisation, multiple episodes and death); adjusted odds ratios (aOR) and 95% confidence interval (CI) were calculated. Categorical variables were presented as total number and percentages and continuous variables as medians and interquartile range (IQR). Comparisons between variables were performed by chi-square test or Fisher exact test for categorical variables and Mann–Whitney U test for continuous variables. Statistical significance was defined as p value < 0.05. Analyses were carried out using Stata 16 and Excel 2019.

## Results

From 1997 to 2019, 905 hospitalised cases with hepatitis E were identified in Spain (Table 1). Median age was 57 years (IQR: 45–69), with up to 84% of patients (n = 757) being 40 years or older; 652 cases (72%) were male and 60 cases (7%) died. Forty-three cases (5%) were admitted on two or more occasions; 32 (74%) were readmitted only once, with a median of 48 days (IQR: 24–126) between episodes. Eleven patients were readmitted two or more times, with a median of 172 days (IQR: 19–275) between the first two episodes and 320 days (IQR: 107–886) between the first and the last episode. The median LOS was 8 days (IQR: 5–16); 540 of 862 (62%) cases with only one admission and 38 of 43 patients with multiple episodes presented a prolonged hospitalisation, i.e. were admitted for more than 6 days. 

**Table 1:** Sociodemographic and clinical characteristics of hospitalised hepatitis E cases, Spain, 1997–2019 (n = 905)

Characteristics	n	%
Sex		
Male	652	72.04
Female	253	27.96
Age group (years)		
< 20	17	1.88
20–39	131	14.48
40–64	449	49.61
≥ 65	308	34.03
Length of stay (days)		
0–3	158	17.46
4–6	169	18.67
≥ 7	578	63.87
Extrahepatic manifestations		
Neurological symptoms	9	0.99
Thrombocytopenia	32	3.54
Acute pancreatitis	8	0.88
Renal manifestation	1	0.11
Severe disease outcomes		
Death^a^	60	6.65
Multiple episodes	43	4.75
Comorbidities		
Chronic liver disease	208	22.98
Diabetes	215	23.76
Alcohol use	160	17.68
Malignant neoplasms	140	15.47
Hepatitis B/C	103	11.38
HIV infection	57	6.30
Transplant	46	5.08

^a^ Information available in 902 patients.

The most prevalent comorbidities were diabetes (24%; n = 215), chronic liver disease (23%; n = 208) and alcohol use (18%; n = 160). One hundred and forty cases (16%) had a malignant neoplasm and 5% (n = 46) had previously received a transplant. The prevalence of HIV infection was 6% (n = 57) and the prevalence of hepatitis B/C was 11% (n = 103). Chronic liver disease and malignant neoplasms were significantly more prevalent in cases with multiple admissions than in those with a single episode and prolonged hospitalisation (42% vs 24%, p value: 0.012; and 37% vs 15%, p value < 0.005; respectively). HIV infection was more frequently among cases with a single episode and prolonged hospitalisation (7% vs 5% with multiple admissions) and the rest of comorbidities were more prevalent in cases with multiple admissions vs a single episode and prolonged hospitalisation (diabetes: 30% vs 27%; alcohol use: 26% vs 19%; hepatitis B/C: 16% vs 10%; transplant: 7% vs 6%), but without significant differences.

### Temporal trends

**Figure 1:**
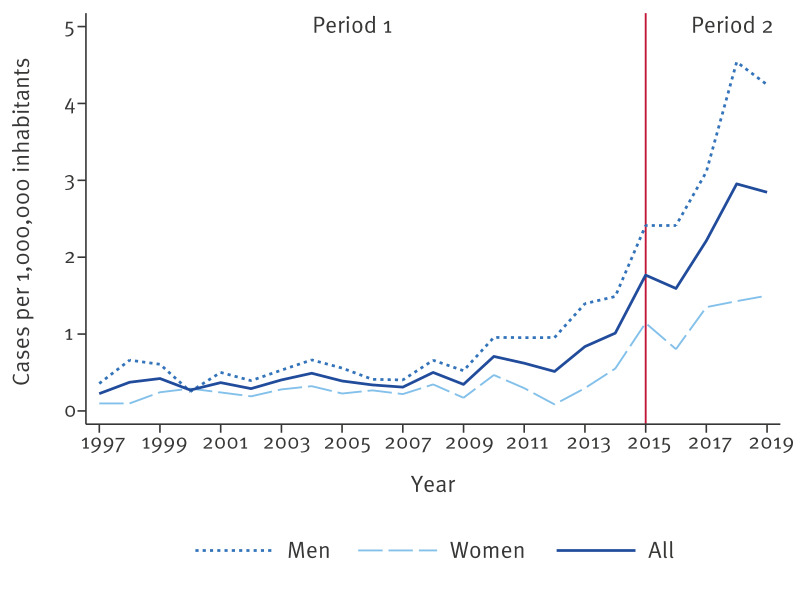
Annual hepatitis E hospitalisation incidence overall and by sex, Spain, 1997–2019 (n = 905)

During Period 1, overall hepatitis E hospitalisation incidence progressively increased from 0.22 cases per 1,000,000 inhabitants in 1997 to 1.77 in 2015; incidence in males was higher than in females during the entire period, except in 2000, when the male-to-female ratio was 0.87 (Figure 1).

**Figure 2:**
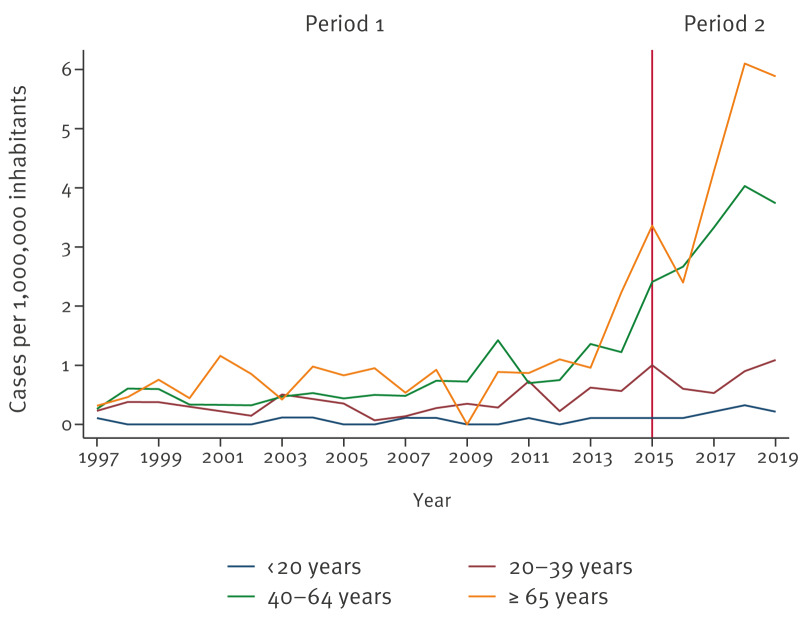
Annual hepatitis E hospitalisation incidence by age group, Spain, 1997–2019 (n = 905)

The population aged above 40 years was slightly more affected than those under this age during the first years of the period (1997–2015), following an upward trend reaching 2.41 and 3.36 hospitalisations per 1,000,000 inhabitants in the 40–64 and ≥ 65 age groups, respectively, in 2015. In the population younger than 20 years, incidence remained less than 0.20, with no cases registered during several years (Figure 2).

**Figure 3:**
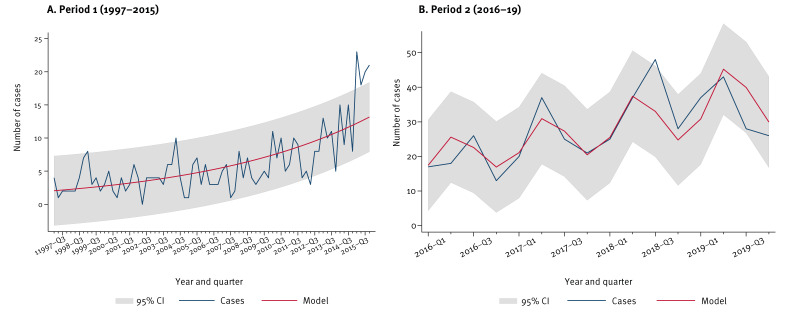
Temporal trends of hospitalised hepatitis E cases, Spain, 1997–2015 (n = 456) and 2016–2019 (n = 449)

CI: confidence interval; Q1: first quarter of each year; Q3: third quarter of each year.

Poisson analysis of Period 1 showed a statistically significant increasing trend in the number of hospitalisations (IRR: 1.03; 95% CI: 1.02–1.03; p value < 0.001). No seasonality was observed between 1997 and 2015 (p value > 0.25) (Figure 3A).

Trends were analysed using a generalised linear model (Poisson regression). The blue line represents the cumulative number of hospitalised hepatitis E cases in each quarter of the year. The red line and shading represent the linear temporal trend with 95% confidence intervals.

Regarding Period 2, annual hospitalisation incidence increased from 2016 to 2018 (with 1.59 and 2.95 cases per 1,000,000 inhabitants, respectively), before declining slightly in 2019 (Figure 1). Males were more affected than females, with a male-to-female ratio ranging from 2.30 in 2017 to 3.18 in 2018 (Figure 2). Compared with the previous period, a greater difference in the hospitalisation incidence between people aged over and under 40 years was observed, with a maximum of 6.10 cases per 1,000,000 in the population aged over 64 years and 4.03 in the 40–64-year age group in 2018. In the age group under 20 years, incidence was lower than 0.35 cases per 1,000,000 during the period (Figure 2). The number of hospitalisations presented a statistically significant increasing trend according to the Poisson regression analysis (IRR: 1.05, 95% CI: 1.03–1.07; p value < 0.001). Furthermore, seasonality was observed (p value < 0.001), with more cases in the second and third quarters of the year (Figure 3B).

A progressive increase both case age and LOS was observed, although the results were not statistically significant (coefficient β = 0.40, p value: 0.096; and coefficient β = 0.09, p value: 0.159, respectively).

### Extrahepatic manifestations

Extrahepatic manifestations were identified in 50 cases (6%). Forty-three cases were male, and the median age was 55 years (IQR: 46–71). The median LOS was 13 days (IQR: 6–21) and 6 cases died. In cases with this type of symptoms, a significantly greater proportion of males and longer LOS were observed comparing with those without extrahepatic symptoms (86% vs 71%, p value: 0.024; and 13 vs 8 days; p value: 0.029; respectively).

Nine patients presented neurological symptoms: three cases with Guillain–Barré syndrome, five cases with neuralgic amyotrophy and one case with mononeuritis multiplex. Cases with these neurological symptoms were significantly younger than cases without them (median age: 47 vs 58 years, p value: 0.031). Thirty-two cases had thrombocytopenia, who presented significantly greater age (median age: 64 vs 57 years, p value: 0.042) and greater LOS (median: 16 vs 8 days, p value: 0.005) than patients without this condition. Eight cases presented acute pancreatitis, with a median age of 45 years and a median LOS of 5 days. Furthermore, one case presented a renal manifestation.

### Severity

Results of multivariate logistic regression of demographic and clinical factors related to length of prolonged hospitalisation, multiple episodes and death are shown in Table 2. Age 50 years and older, transplant and HIV infection were independently associated with prolonged hospitalisation, while chronic liver disease and malignant neoplasm were independently associated with multiple episodes. Age 50 years and older, chronic liver disease, HIV infection and hepatitis B/C were independently associated with death.

**Table 2:** Demographics and clinical comorbidities of cases with hepatitis E associated with severe outcomes, Spain, 1997–2019 (n = 905)

Characteristics	Prolonged hospitalisation			Multiple episodes			Death^a^		
	aOR	95% CI	p value	aOR	95% CI	p value	aOR	95% CI	p value
Demographics									
Age group (ref. < 50 years)									
≥ 50 years	2.01	1.47–2.75	< 0.001	2.12	0.87–5.21	0.100	2.43	1.14–5.17	0.021
Sex (ref. females)									
Males	1.24	0.90–1.70	0.185	0.83	0.41–1.71	0.620	0.72	0.39–1.33	0.290
Comorbidities									
Chronic liver disease (ref. no)									
Yes	1.32	0.92–1.92	0.136	2.03	1.01–4.07	0.047	4.29	2.38–7.73	< 0.001
Diabetes (ref. no)									
Yes	1.42	0.99–2.03	0.055	1.12	0.56–2.26	0.746	0.71	0.36–1.39	0.316
Alcohol use (ref. no)									
Yes	1.18	0.78–1.78	0.444	1.24	0.54–2.84	0.612	0.71	0.37–1.40	0.324
Malignant neoplasm (ref. no)									
Yes	1.12	0.75–1.68	0.582	2.98	1.51–5.85	0.002	0.80	0.38–1.70	0.562
Hepatitis B/C (ref. no)									
Yes	0.72	0.46–1.12	0.124	1.81	0.74–4.43	0.195	2.11	1.01–4.42	0.047
HIV infection (ref. no)									
Yes	2.44	1.30–4.59	0.006	1.04	0.21–5.19	0.957	3.00	1.07–8.44	0.038
Transplant (ref. no)									
Yes	2.13	1.02–4.44	0.045	1.06	0.30–3.80	0.928	1.50	0.48–4.70	0.486

aOR: adjusted odds ratio; CI: confidence interval; ref.: reference.

^a^ Information available in 902 patients.

OR and 95% CI were adjusted for age group, sex, chronic liver disease, diabetes, alcohol use, malignant neoplasm, transplant, HIV infection and hepatitis B/C.

### Geographical distribution

**Figure 4:**
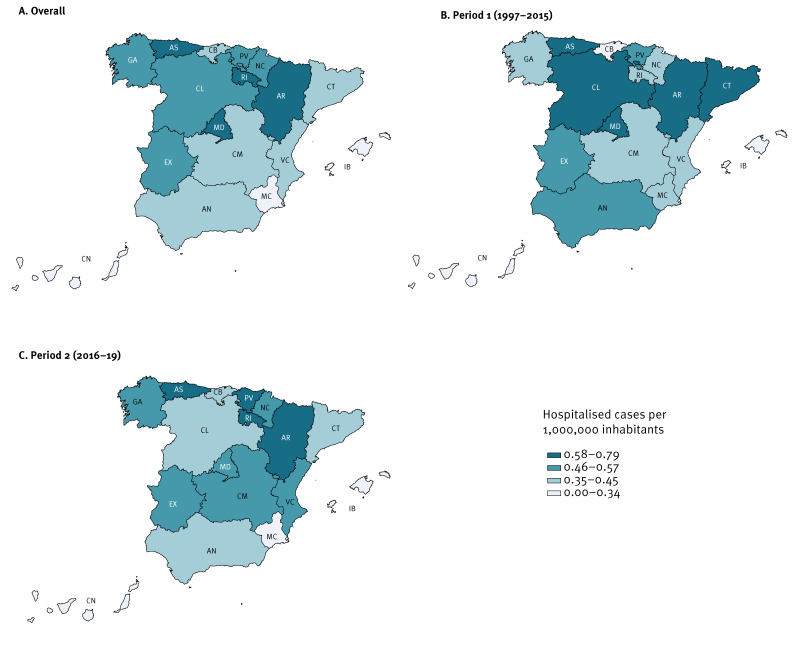
Geographical distribution of hospitalised hepatitis E cases, Spain, 1997–2019 (n = 881)

AN: Andalusia; AR: Aragon; AS: Asturias; CN: Canary Islands; CB: Cantabria; CE/ML: Ceuta and Melilla; CM: Castile-La Mancha; CL: Castile and Leon; CT: Catalonia; EX: Extremadura; GA: Galicia; IB: Balearic Islands; RI: La Rioja; MD: Community of Madrid; MC: Region of Murcia; NC: Navarre; PV: Basque Country; VC: Valencian Community.

Information about region of residence was available in 881 patients (97.34%). A clear pattern in the geographical distribution of the disease was not identified, as the regions with the highest incidences presented differences in terms of population size and sociodemographic characteristics. The greatest hospitalisation incidences were observed in regions located in the northern and centre of the country: La Rioja (2.02 cases/1,000,000 inhabitants), Aragon (1.66), Community of Madrid (1.19) and Asturias (1.15) (Figure 4A). Nevertheless, differences were observed between periods: in Period 1, the highest incidences of hospitalisation were found in Community of Madrid (0.79) followed by Castile and Leon (0.74) and Asturias (0.64) (Figure 4B), but in Period 2, regions located mainly in the north of the country had higher incidence: La Rioja (9.58), Aragon (6.64), Basque Country (3.91) and Asturias (3.65) (Figure 4C).

## Discussion

Our study, which encompasses more than 20 years and close to 1,000 hepatitis E hospitalised cases, provides valuable data on the characteristics of cases with this disease in Spain, such as comorbidities associated with greater severity and death. This information has so far not been available at national level as the notification of hepatitis E is not mandatory in the country. Overall, we found that most of the cases were men aged over 40 years, whereas cases in people under 20 years were rare, as reported in other neighbouring countries [13,14] and in a seroprevalence study carried out in Spain, in which the lowest IgG-HEV prevalence was found in people under 20 years (5%) and the highest in the 70–80-year age group (39%) [15]. The long LOS (median: 8 days) may result in costly medical expenses and may be related to adverse clinical outcomes.

Since 1990, an increase in the number of HEV infection cases in world regions with high socio-demographic index has been observed, especially in the population older than 40 years [16]. We observed a statistically significant increasing trend in the annual incidence of hepatitis E hospitalisation in Spain in both Period 1 and Period 2. This upward trend may be related to a better awareness among clinicians and an improvement in the diagnosis of the infection, i.e. introducing routine HEV testing into laboratory protocols for people with impaired liver enzymes or symptoms consistent with acute hepatitis [7,17]. Nevertheless, during and after the COVID-19 pandemic, no clear upward trend in the number of hospitalised cases of hepatitis E in Spain has been observed. According to aggregate data available on the CMBD website, 153, 183 and 186 hospitalised cases were reported in the years 2020, 2021 and 2022, respectively [18]. We observed a seasonal pattern in Period 2, with more cases detected in the months of April to September. This higher incidence of the disease in the spring and summer months has also been found in other European studies [19,20].

Possible extrahepatic manifestations were identified in around 6% patients, who had a longer LOS and higher incidence of death, which may point to a more difficult clinical management of these patients. Nine cases presented neurological symptoms, and in five of these, the neurological condition was encoded as the primary diagnosis, possibly showing than these symptoms were the first clinical manifestations of the infection. The prevalence of neurological symptoms (1%) in this study is lower than that observed in other European case series, in which patients with this type of symptomatology were significantly younger, a characteristic which was also observed in our study [21,22]. Hepatitis E virus infection has also been related to dementia; in a case–control study conducted in Spain, a statistically significant association between the prevalence of antibodies against HEV in serum and neurological disorders was observed, which may point out an underestimated contribution of HEV infection to central nervous system disease [23].

A wide range of comorbidities have been identified in our study population, with diabetes, chronic liver disease, and alcohol use being the most prevalent. Six percent of cases were infected with HIV; several studies suggest there is no evidence of higher prevalence of antibodies against HEV in serum in this specific population group [24-27]. Around 7% of our patients died, which equals a case fatality rate higher than those observed in other series of hospitalised hepatitis E patients [28]. Factors independently associated with death were age over 50 years, chronic liver disease, HIV infection and hepatitis B/C. Chronic liver disease, including chronic hepatitis B, has been previously related to severe hepatitis E, with HEV superinfection being a risk factor for acute-on-chronic liver failure, particularly in patients with cirrhosis [29-32]. This condition has also been associated with severe disease in hepatitis A hospitalised patients [33]. A total of 43 patients presented multiple admissions, with an interval between admissions ranging from several weeks in those readmitted only once (which may be part of the same acute process, since according to the literature, symptoms last up to 6 weeks [1]), to months in those with three or more episodes, which may indicate either chronic infections or reinfections. In our study, malignant neoplasm was independently associated with multiple episodes, and chronic hepatitis E infection has been described in patients with immunosuppression [34,35].

An increased risk of hepatitis E infection has been associated with occupational exposure, i.e. agriculture and livestock farming [36,37] or forestry workers [38], and with dietary habits i.e. the consumption of processed pork products such as bacon, cured pork meats and pigs’ liver [39]. These factors, in conjunction with possible differences in the diagnostic capabilities of hospital microbiology laboratories, may also explain the observed differences by regions within the country. Further studies are needed to determine the role of these environmental or social factors in the transmission of hepatitis E virus, with cross-disciplinary collaboration of human, animal, food and environmental public health authorities under a One Health approach. In addition, whole genome sequencing techniques can also help characterise the virus and improve understanding of its routes of transmission.

Several limitations have been identified. Firstly, our study reflects only part of the clinical spectrum of the disease, e.g. hospitalisations including severe cases, while according to the literature most HEV infections are asymptomatic or mild. Secondly, data quality obtained from the CMBD is quite dependent on the precision of diagnostic codification in the hospital discharge reports. Furthermore, some relevant information about the patients, such as symptoms, clinical evolution, treatment, laboratory results, microbiological tests used in the diagnosis of hepatitis E (PCR and/or serology), HEV genotype/subtype and origin of the infection (autochthonous/imported) were not available. Thirdly, during the study period, a change in the International Classification of Diseases edition occurred (from ICD-9 to ICD-10), consequently the diagnostic codes of hepatitis E, as well as the diagnostic codes used to identify comorbidities and extrahepatic manifestations, differed between the temporal periods (1997–2015 and 2016–19). Moreover, in Period 2, with the establishment of the new CMBD, patients from other types of healthcare facilities in addition to acute care hospitals were also included. Nevertheless, coding within periods was stable, and temporal trends were analyses separately using linear Poisson model. Given the modest incidence of the disease, a low number of cases were available for the Poisson trend analyses, so the results should be interpreted with caution. Finally, individualised data from the CMBD need to be consolidated, so they are available with some delay. This fact, along with the low incidence of the disease and the possible disruption in the data series during the COVID-19 pandemic, led us to only analyse data up to and including the year 2019.

## Conclusions

Our study shows that hepatitis E hospitalisations are increasing in Spain, presenting more severity in patients with conditions such as older age, chronic hepatic diseases and HIV infection. The study highlights the usefulness of complementary sources of information and electronic records, such as hospital admissions, for epidemiological surveillance. Further studies are needed to assess the seasonality, geographical distribution and impact of the disease and to guide public health actions for prevention and control. 

## Supplementary Data

466000 Supplement
