# Supplementary material for: Increasing trends in hepatitis E hospitalisations in Spain, 1997 to 2019
Source: Euro Surveill. 2024 Oct 24;29(43):2400118. doi: 10.2807/1560-7917.ES.2024.29.43.2400118 (PMC11513759; doi:10.2807/1560-7917.ES.2024.29.43.2400118)
Supplement: Supplement [file 24-00118_GUERRERO_Supplement.pdf]

This supplementary material is hosted by *Eurosurveillance* as supporting information alongside the article "Increasing trends in hepatitis E hospitalisations in Spain, 1997 to 2019" on behalf of the authors who remain responsible for the accuracy and appropriateness of the content. The same standards for ethics, copyright, attributions and permissions as for the article apply. *Eurosurveillance* is not responsible for the maintenance of any links or email addresses provided therein.

Supplementary table S1. ICD-9 and ICD-10 codes used to define hepatitis E infection, extrahepatic manifestations and comorbidities.

Variable	ICD-9 codes	ICD-10 codes
Hepatitis E	070.43 Hepatitis E with hepatic coma)<br>070.53 Hepatitis E without mention of hepatic coma	B17.2 Acute hepatitis E
Neuralgic amiotrophy	353.5 Neuralgic amyotrophy	G54.5 Neuralgic amyotrophy
Guillain-Barre síndrome	357.0 Acute infective polyneuritis	G61.0 Guillain-Barre syndrome
Mononeuritis multiplex	354.5 Mononeuritis multiplex	G58.7 Mononeuritis multiplex
Thrombocytopenia	287.49 Other secondary thrombocytopenia<br>287.5 Thrombocytopenia, unspecified	D69.59 Other secondary thrombocytopenia<br>D69.6 Thrombocytopenia, unspecified
Acute pancreatitis	577.0 Acute pancreatitis	K85 Acute pancreatitis

Renal manifestations	581.0 Nephrotic syndrome with lesion of proliferative glomerulonephritis<br>581.1 Nephrotic syndrome with lesion of membranous glomerulonephritis<br>581.2 Nephrotic syndrome with lesion of membranoproliferative glomerulonephritis<br>583.0 Nephritis and nephropathy, not specified as acute or chronic, with lesion of proliferative glomerulonephritis<br>583.1 Nephritis and nephropathy, not specified as acute or chronic, with lesion of membranous glomerulonephritis<br>583.2 Nephritis and nephropathy, not specified as acute or chronic, with lesion of membranoproliferative glomerulonephritis<br>583.9 Nephritis and nephropathy, not specified as acute or chronic, with unspecified pathological lesion in kidney	N02.2 Recurrent and persistent hematuria with diffuse membranous glomerulonephritis<br>N02.3 Recurrent and persistent hematuria with diffuse mesangial proliferative glomerulonephritis<br>N02.4 Recurrent and persistent hematuria with diffuse endocapillary proliferative glomerulonephritis<br>N04.2 Nephrotic syndrome with diffuse membranous glomerulonephritis<br>N04.3 Nephrotic syndrome with diffuse mesangial proliferative glomerulonephritis<br>N04.4 Nephrotic syndrome with diffuse endocapillary proliferative glomerulonephritis<br>N05.2 Unspecified nephritic syndrome with diffuse membranous glomerulonephritis<br>N05.3 Unspecified nephritic syndrome with diffuse mesangial proliferative glomerulonephritis<br>N05.4 Unspecified nephritic syndrome with diffuse endocapillary proliferative glomerulonephritis<br>N05.5 Unspecified nephritic syndrome with diffuse mesangiocapillary glomerulonephritis

		N02.8 Recurrent and persistent hematuria with other morphologic changes
Chronic liver disease	571 Chronic liver disease and cirrhosis<br>572.8 Other sequelae of chronic liver disease	K70 Alcoholic liver disease<br>K72.1 Chronic hepatic failure<br>K72.9 Hepatic failure, unspecified<br>K73 Chronic hepatitis, not elsewhere classified<br>K74 Fibrosis and cirrhosis of liver<br>K76.0 Fatty (change of) liver, not elsewhere classified

Diabetes	249 Secondary diabetes mellitus<br>250 Diabetes mellitus	E08 Diabetes mellitus due to underlying condition<br>E09 Drug or chemical induced diabetes mellitus<br>E10 Type 1 diabetes mellitus<br>E11 Type 2 diabetes mellitus<br>E13 Other specified diabetes mellitus
Alcoholism	291 Alcohol-induced mental disorders<br>303 Alcohol dependence syndrome<br>305.0 Nondependent alcohol abuse<br>571.0 Alcoholic fatty liver<br>571.1 Acute alcoholic hepatitis<br>571.2 Alcoholic cirrhosis of liver<br>571.3 Alcoholic liver damage, unspecified<br>V11.3 Personal history of alcoholism	F10.1 Alcohol abuse<br>F10.2 Alcohol dependence<br>F10.9 Alcohol use, unspecified<br>K70 Alcoholic liver disease<br>Z71.41 Alcohol abuse counseling and surveillance<br>Y90 Evidence of alcohol involvement determined by blood alcohol level
Malignant neoplasm	140-239 Neoplasms<br>V10 Personal history of malignant neoplasm	C00-C96 Malignant neoplasms<br>Z85 Personal history of malignant neoplasm
Hepatitis B and/or C	070.2 Viral hepatitis b with hepatic coma<br>070.3 Viral hepatitis b without mention of hepatic coma<br>070.41 Acute hepatitis C with hepatic coma<br>070.44 Chronic hepatitis C with hepatic coma<br>070.51 Acute hepatitis C without mention of hepatic coma<br>070.54 Chronic hepatitis C without mention of hepatic coma<br>070.7 Unspecified viral hepatitis C	B16 Acute hepatitis B<br>B17.1 Acute hepatitis C<br>B18.0 Chronic viral hepatitis B with delta-agent<br>B18.1 Chronic viral hepatitis B without delta-agent<br>B18.2 Chronic viral hepatitis C<br>B19.1 Unspecified viral hepatitis B<br>B19.2 Unspecified viral hepatitis C

HIV infection	042 Human immunodeficiency virus [HIV] disease	B20 Human immunodeficiency virus [HIV] disease
Transplant	996.8 Complications of transplanted organ<br>V42 Organ or tissue replaced by transplant	T86 Complications of transplanted organs and tissue<br>Z48.2 Encounter for aftercare following organ transplant<br>Z94 Transplanted organ and tissue status
